# The Marine-Derived Fungus *Clonostachys rosea*, Source of a Rare Conjugated 4-Me-6*E*,8*E*-hexadecadienoic Acid Reducing Viability of MCF-7 Breast Cancer Cells and Gene Expression of Lipogenic Enzymes

**DOI:** 10.3390/md13084934

**Published:** 2015-08-06

**Authors:** Ana Camila Dos Santos Dias, Nicolas Ruiz, Aurélie Couzinet-Mossion, Samuel Bertrand, Muriel Duflos, Yves-François Pouchus, Gilles Barnathan, Hassan Nazih, Gaetane Wielgosz-Collin

**Affiliations:** 1 Faculty of Pharmacy, University of Nantes, MMS, 9, Rue Bias, 44000 Nantes, France; E-Mails: acamiladias@gmail.com (A.C.D.S.D.); nicolas.ruiz@univ-nantes.fr (N.R.); aurelie.couzinet-mossion@univ-nantes.fr (A.C.-M.); samuel.bertrand@univ-nantes.fr (S.B.); yves-francois.pouchus@univ-nantes.fr (Y.-F.P.); gilles.barnathan@univ-nantes.fr (G.B.); 2 Faculty of Pharmacy, University of Nantes, IICiMed, 9 Rue Bias, 44000 Nantes, France; E-Mail: muriel.duflos@univ-nantes.fr

**Keywords:** *Clonostachys rosea*, fatty acids, conjugated fatty acid, lipids, cancer, anti-carcinogenic, MCF-7, lipogenesis, acetyl-CoA carboxylase, fatty acid synthase

## Abstract

A marine-derived strain of *Clonostachys rosea* isolated from sediments of the river Loire estuary (France) was investigated for its high lipid production. The fungal strain was grown on six different culture media to explore lipid production changes. An original branched conjugated fatty acid, mainly present in triglycerides and mostly produced when grown on DCA (23% of total fatty acid composition). It was identified as 4-Me-6*E*,8*E*-hexadecadienoic on the basis of spectroscopic analyses. This fatty acid reduced viability of MCF-7 breast cancer cells in a dose dependent manner (up to 63%) at physiological free fatty acid human plasma concentration (100 μM). Reduction of gene expression of two lipogenic enzymes, the acetyl CoA carboxylase (ACC) and the fatty acid synthase (FAS) was evaluated to explore the mechanisms of action of 4-Me-6*E*,8*E*-16:2 acid. At 50 μM, 50% and 35% of mRNA gene expression inhibition were observed for ACC and FAS, respectively.

## 1. Introduction

Various oleaginous yeasts and filamentous fungi have been recognized for their production of lipids. In fact, they grow quickly on different media, have a short life cycle, require less maintenance and produce significant amounts of oil [1]. The lipid content of these microorganisms may vary depending on the conditions of cultivation such as pH, temperature, ratio between carbon and nitrogen available in the medium [2]. For instance, under conditions of nitrogen stress, intracellular lipid production of yeast can increase significantly depending on the strain [3]. Over the years, the fatty acid (FA) profiles present in oleaginous microorganisms were explored. FA composition of a several fungi belonging to Oomycetes, Zygomycetes, Ascomycetes, and Basidiomycetes have been studied. It has been demonstrated that they are a source of various FA, however their general composition stay constant but with different proportions. The most common and abundant FA are palmitic acid (16:0), stearic acid (18:0), oleic acid (9-18:1), and linoleic acid (9,12-18:2) [4,5,6]. Furthermore, some studies also showed that most lower fungi (Chytridiomycetes, Oomycetes, and Zygomycetes) are characterized by the significant presence of polyunsaturated fatty acids (PUFA) unlike some species of higher fungi (Ascomycetes and Basidiomycetes) which produce only α-linolenic acid (9,12,15-18:3), the precursor of the biosynthetic pathway of ω3 FA, known as essential FA. Higher fungi can also produce essential PUFA such as arachidonic acid (20:4 ω6) or docosahexaenoic acid (22:6 ω3) but in lower proportions [4,5,6]. Some studies have demonstrated that certain species of Ascomycetes (yeasts and molds) could produce essential FA such as γ-linoleic acid (GLA) and ω6 [7], produced by the unsaturation of linoleic acid. This production is highly dependent on the source of carbon used in the culture medium.

Studies focusing on FA from marine-derived fungi are still rare [8,9,10,11]. Therefore, these marine microorganisms represent an alternative FA production approach for a use in human health and nutrition.

In a previous study, we have shown that fungal lipid production by Agar surface fermentation is an easy to operate process and could be used for further screening of marine-derived fungi [8]. In this context, our attention focuses on a marine-derived strain of *Clonostachys rosea* (MMS1090), a filamentous fungus isolated from marine sediments of the river Loire estuary (France), which was detected as a high lipid producer. *C. rosea* (Ascomycete, syn. *Gliocladium roseum*: Teleomorph *Bionectria ochroleuca*) [12], is known for its high lipid production [13]. In addition, it was also reported to produce volatile compounds, major components of biodiesel [14].

To optimize the lipid production a “One Strain Many Compounds” (OSMAC) approach was performed on six culture media [15] and FA composition was determined by gas chromatography coupled to mass spectrometry (GC-MS). An unusual conjugated fatty acid (CFA) was purified and identified based on spectroscopic data. Because some CFA (such as conjugated *E*9,*E*11-octadecadienoic acid) have already shown inhibitory effects on mammary cancerous cell (MCF-7) proliferation [16] this unusual CFA was evaluated for its anti-proliferative activity on MCF-7 breast cancer cells. In addition, as aberrant lipogenesis in cancer cells is mediated by increased expression and activity of acetyl CoA carboxylase (ACC) and fatty acid synthase (FAS) [17], the mechanism of action of this rare CFA was further explored on these two lipogenic enzymes.

## 2. Results and Discussion

Lipids from microorganisms have a real importance in human health, once the FA composition studied, they can be tested against several diseases. However, the lipid composition of *Clonostachys rosea* (*C. rosea*) was not yet investigated in different culture media. It could be of interest in the pharmacological field, including research of new molecules.

### 2.1. Lipid and Fatty Acid Composition of C. rosea—OSMAC Approach

To optimize the lipid production of the marine-derived strain of *C. rosea* MMS1090, an OSMAC approach was performed on six culture media. FA profiling was determined by GC-MS on the six extracts (Table 1). Changes of lipid production by the strain were observed and media composition compared according to the amount of supplemented sugar (see Experimental Section for the amount of supplemented sugar). An unusual CFA was observed.

**Table 1:** Total lipid content (TL % dw) and major fatty acid composition (% total FA) of *C. rosea* on six different culture media.

Culture Medium	Lipid Content (%, dw)	Fatty Acids (Mean ± SD) (% Total FA)				
		16:0	4-Me-6,8-16:2	18:2 and 18:1	18:0	Others FA *
PDA	12.9 ± 0.3	16.1 ± 0.9	8.0 ± 3.0	55.0 ± 4.0	10.0 ± 2.0	12.0 ± 7.0
MES	29.7 ± 0.4	20.0 ± 5.0	9.0 ± 2.0	52.5 ± 13.5	8.0 ± 3.0	10.0 ± 5.0
CYA	8.7 ± 0.8	15.7 ± 0.5	14.0 ± 1.0	60.0 ± 17.0	6.0 ± 0.1	4.0 ± 3.0
YES	30.7 ± 0.4	14.0 ± 2.0	11.0 ± 1.0	64.0 ± 6.0	6.0 ± 1.0	4.0 ± 2.0
DCA	14.3 ± 0.4	16.0 ± 1.0	23.0 ± 0.8	44.6 ± 4.7	5.0 ± 1.0	10.0 ± 6.0
MEA	14.9 ± 0.2	17.5 ± 0.4	8.0 ± 2.0	57.0 ± 2.0	9.0 ± 0.5	9.0 ± 6.0

* % of total fatty acid content ≤3%.

FA compositions observed indicated the ability of this strain to use the different available nutrients provided in the culture media leading to some differences in FA production. The highest lipid contents were obtained on YES and MES media, 30.7% and 29.7%, respectively. These high values could be explained by the high sucrose content of these culture media (150 g sucrose/L) which would engage the lipid cells accumulation. In fact, under suitable culture conditions, some microorganisms may convert the carbon sources from several substrates into storage lipid [18,19]. The stocking rate for fungi could reach 70% of the cell biomass [20,21]. Regarding the other culture media, the values for the production of lipid are almost two times lower.

Each extract was saponified and the total FA composition was determined by GC-MS analysis. Finally, all FA were identified by MS^EI^ spectra investigation and the five major FA are presented in Table 1. Analysis of fatty acid methyl esters (FAME) by GC-MS is used to define the length of the carbon chain, to classify FA by their number of double bonds or ramifications, and estimate their relative proportion. Analysis of *N*-acyl pyrrolidide derivatives (NAP) allows positioning the double bonds on the unsaturated FA. Therefore, they become complementary to analyze FA composition. All the extracts contained these five major FA representing between 88% and 96% of total FA. However, a total of 31 FA were identified in this strain, corresponding to 14 to 24 carbon FA. Four of them, 16:0, 18:0, 9-18:1 and 9,12-18:2 are the most common FA described in oleaginous microorganisms [4,8,9,10]. Among the different cultures analyzed, a rare FA was observed mainly when grown on DCA medium (23% of total lipid). Providing some amount of amino acids in the culture medium seems to promote its production. Indeed, DCA medium contains 10 g/L of hydrolysed casein and CYA and YES media provide yeast extract which are rich in amino acids. These three culture media of *C. rosea* lead to the production of 23%, 14% and 11% of this particular FA, respectively.

In order to know the repartition of observed FA in the different lipid classes, DCA extract was partitioned on an open silica gel column leading to fractions corresponding to the different lipid classes. After saponification and esterification, FA composition was analyzed by GC-MS for each fraction (Table 2). This *C. rosea* oleaginous strain extract contained a high triglyceride (TG) content (84%) composed mainly of unsaturated FA (58%). Few studies on the separation of lipid classes were made on fungi; usually neutral lipids are the major ones when fungi are cultivated on solid medium [8]. Their proportion varies according to the lipid class and certain FA are present in only a specific class, such as hydroxylated FA observed only in the phospholipids.

**Table 2:** Fatty acid composition to different lipid classes for *C. rosea* on DCA culture medium.

Lipid production		Main FA Composition (% total FA)		
Lipid Classes	% of Total Lipids	Saturated FA *	Unsaturated FA *	4-Me-6,8-16:2
Triglycerides	84 ± 7	28 ± 2	58 ± 7	20 ± 4
Glycolipids	4 ± 2	46 ± 3	44 ± 2	5 ± 1
Phospholipids	12 ± 6	29 ± 3	64 ± 6	3 ± 1

Also identified (≤3%): 14:0, 15:0, 9-16:1, 4-Me-6-16:1, 6,8-16:2, 17:0, 4-Me-6,8-16:2 (isomer A), 4-Me-6,8-16:2 (isomer B), cj-17:2, 9,11-18:2, 10,12-18:2, cj-18:3, 19:2, cj-19:2, 10,14-20:2, 10-20:1, 11-20:1, 20:0, 21:0, 12-22:1, 22:0, 23:0, 2-OH-22:0**, 24:1, 24:0, 2-OH-24:0**. cj, conjugated; * only major FA for saturated (16:0 and 18:0) and unsaturated (4-Me-6,8-16:2 ; 18:1 and 18:2); ** only presents in phospholipids.

**Figure 1:**
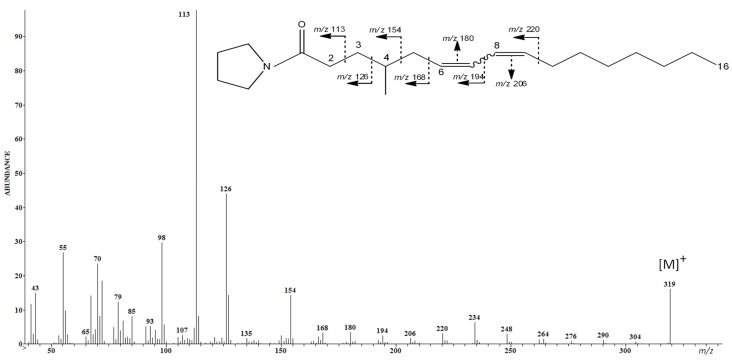
NAP spectrum of the 4-Me-6,8-16:2 acid (isomer C) produced by *Clonostachys rosea*.

Three isomers (A, B, and C) of the unusual CFA, the 4-Me-6,8-16:2 acid, have been identified based on their chromatographic behavior, the analysis of their FAME and their NAP spectra. Figure 1 shows the NAP spectrum of the isomer C. A molecular ion peak at *m*/*z* 319 [M]^+^ confirmed the heptadecadienoic structure. A diminished peak corresponding to the fragment at *m*/*z* 140 [M-179]^+^ flanked by an elevated peak corresponding to the fragment at *m*/*z* 154 [M-165]^+^ indicated a methyl branch at the fourth carbon. The conjugated diene system (Δ6,8) was located at C6-C7, and C8-C9 where a difference of 12 in *m*/*z* ratios was observed between each couple of those carbons. Isomers A and B of 4-Me-6,8-16:2 acid were present in low amounts, less than 2% of total FA. The third-one was present in higher amount in TG (20%), which implies that it could be a storage FA [22].

The presence of the unusual 6,8-16:2 acid was also detected in low amount (1.3% of total FA). This CFA differs from the three previous isomers of 4-Me-6,8-16:2 acid by the lack of methyl branch on C-4. A possible biosynthetic link could be suggested between these CFA. Nevertheless, the biosynthetic pathway involving the production of the three 4-Me-6,8-16:2 isomers remain unknown. Biosynthesis of CFA is well documented in bacteria from rumen origin in relation to the production of conjugated linoleic acids (CLA). CLA are produced by isomerization of linoleic acid or by Δ9-desaturation of *trans*-11-octadecenoic acid (*trans*-vaccenic acid) [23,24]. In fungi, CLA are mainly related to the Δ9-desaturation of *trans*-vaccenic acid [24]. A possible way of biosynthesis of the 4-Me-6,8-16:2 acid could be a desaturation of the 4-Me-6-16:1 which was also detected in low amounts (less than 1% of TFA). In addition, it could be suggested that these methyl-branched CFA arise from a mixed isoprenoid/acetate biogenesis.

The presence of branched and conjugated FA in a fungus is therefore unexpected and remains rare.

### 2.2. Characterization of 4-Me-6-8-Hexadecadienoic Acid Structure (Isomer C)

For structure identification of the isomer C of 4-Me-6,8-16:2 acid, its isolation was necessary. For that purpose, cultures of *C. rosea* MMS1090 were performed on nine DCA-containing Petri dishes to produce sufficient amount. After fungal biomass extraction, the crude lipid extract (4.4 g) was saponified leading to free total FA (3.5 g). Enrichment and purification of the 4-Me-6,8-16:2 was performed by urea fractionation followed by consecutive silver ion column chromatography and silver ion HPLC. The final fraction (100 mg) contained the 4-Me-6,8-16:2 methyl ester with a purity of 98% (according to GC-MS). Proton NMR analysis confirmed the structure of the CFA (Table 3), the spectrum being consistent with data previously described in the literature [25]. The ^13^C NMR spectra (Table 3) showed the signals for 18 carbons and the DEPT 135 spectra distinguished a switch between C-4 and C-10 signals in comparison to previously reported spectra [25]. Starting from the proton of C-5 or C-10, all the ^1^H and ^13^C NMR signals of the conjugated double bonds were assigned by using the homonuclear correlation spectroscopy (COSY), and heteronuclear single quantum correlation (HSQC).

**Table 3:** ^1^H and ^13^C NMR spectroscopic data for FAME of 4-Me-6,8-16:2 (structure and atom number in Scheme 1).

Positions	δ_C_	δ_H_ (Integral, Mult., *J* = Hz)	
1	174.4	-	-
2	31.9	1.33	(1H, m)
		2.33	(1H, m)
3	31.8	1.33	(1H, m)
		2.33	(1H, m)
4	32.9	1.48	(1H, m)
5	39.8	1.99	(1H, dt, 7.2, 6.8)
		2.05	(1H, dt, 7.2, 6.8)
6	129.8	5.54	(1H, m)
7	130.1	5.99	(1H, m)
8	132.03	5.99	(1H, m)
9	132.9	5.54	(1H, m)
10	32.6	2.05	(2H, m)
11	29.2	1.44	(2H, m)
12	31.4	1.33	(1H, m)
		1.70	(1H, m)
13	29.4	1.33	(2H, m)
14	29.1	1.33	(2H, m)
15	22.6	1.33	(2H, m)
16	14.1	0.86	(3H, t, 6.8)
17	19.1	0.88	(3H, d, 6.4)
–OCH_3_	51.5	3.66	(3H, s)

Taken in CDCl_3_ at 400 MHz for ^1^H and at 100 MHz for ^13^C.

**Scheme 1:**
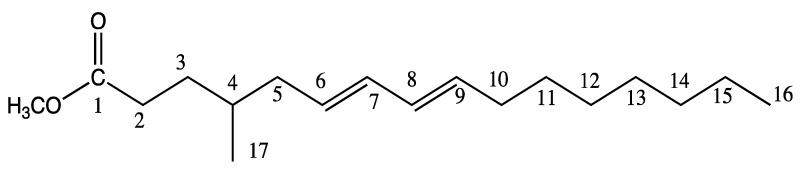
Chemical structure of 4-Me-6*E*,8*E*-16:2 methyl ester.

Infrared spectroscopy analysis was performed to determine the configuration of the diene system. A characteristic band of strong intensity is observed at 987 cm^−1^, corresponding to the stretching vibration of CH *trans.* In addition, no absorbance at 700–750 cm^−1^ corresponding to a *cis* stretching vibration was observed*.* Therefore, the structure of this unusual CFA is 4-Me-6*E*,8*E*-16:2.

To our knowledge conjugated 4-Me-16:2 acids were rarely described in literature. For instance, there is only one description of the 4-Me-6,8-16:2 acid, identified in a cytochalasan derivative and corresponding to the trans Δ6,8 isomer [25]. This FA was described from the mushroom *Microporellus subsessilis*, a Polyporaceae (Basidiomycota) collected in Indonesia, taxonomically distant of *C. rosea*. Moreover, it is the first report of the production of such CFA from a filamentous fungi belonging to the Ascomycota and isolated from the marine environment. The fact that this CFA is rare and not universal in microorganisms makes it interesting as a taxonomic biomarker.

Due to the presence of conjugated diene system, CFA have attracted particular attention as active compounds due to their remarkable biological activities. They were reported to possess anti-carcinogenic, immune modulator, anti-diabetic, anti-obesity, antithrombotic, and anti-atherogenic activities [16,26,27,28,29,30] but also antimicrobial ones [31]. The main natural sources of CFA correspond to ruminant milk, algae, and seed oils of several plants [23,26]. On the past ten years, numerous investigators have extensively studied the beneficial role of CLA and ω3 FA against tumor growth as well as tumor types both *in vitro* and *in vivo*. The protective effect of CLA against tumors is well documented [16]. In addition, some nutritional trials showed that CLA supplemented diet lead to their incorporation in the mammary gland in a group of women [32,33]. This can easily suggest that CFA could be used as oil supplements, in pill or liquid form, to prevent or reduce mammary cancer. However, despite the considerable investigation on CLA, no study is available on the role of other CFA such as the 4-Me-6*E*,8*E*-16:2. Fungi like *C. rosea* seem to represent an alternative renewable source of those remarkable compounds.

### 2.3. 4-Me-6E,8E-Hexadecadienoic Acid Reduces Viability of Human MCF-7 Breast Cancer Cells

**Figure 2:**
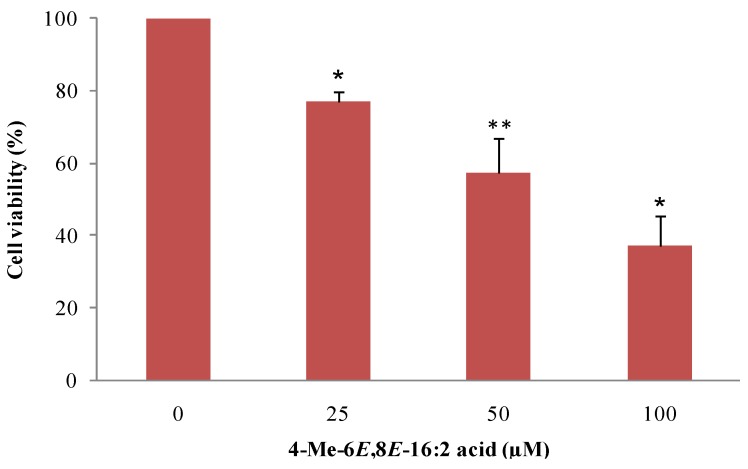
Effect of 4-Me-6*E*,8*E*-16:2 acid on proliferation of MCF-7 cancer cell line. MCF-7 cells were treated with 4-Me-6*E*,8*E*-16:2 acid at 25 μM, 50 μM and 100 μM for 48 h (*n* = 3 experiments in triplicates). * *p* < 0.001 *vs.* control; ** *p* < 0.01 *vs.* control (*t*-test).

The original structure of this rare FA, with a conjugated diene system and a methyl branch, suggest potential biological activity [16,29,34,35]. To evaluate its anti-proliferative activity, viability of MCF-7 breast cancer cell line after treatment with 4-Me-6*E*,8*E*-16:2 acid was assessed using MTT assays. The dose level used, ranging from 0 to 100 μM, was consistent with previous studies with PUFA and CLA and closed to the physiological range concentration normally observed in human plasma (10–80 μM) [16,36,37,38,39]. The 4-Me-6*E*,8*E*-16:2 acid reduced the viability in a dose dependent manner (Figure 2). After 48 h, significant growth inhibitions of 43% and 63% (*p* < 0.05) were observed at 50 μM and 100 μM, respectively.

### 2.4. 4-Me-6E,8E-Hexadecadienoic Acid Reduces Gene Expression of Lipogenic Enzymes

Numerous studies have demonstrated that cancer cell proliferation is associated with hyperactivity of lipogenesis [40,41,42,43,44] manifested by high expression of the lipogenic enzymes. The alteration of the activity of the key lipogenic enzymes (ACC and FAS) is critical for the development of the malignant phenotypes [41,45]. Furthermore, overexpression of FAS correlates with poor prognosis in several types of human malignancies [46]. In addition, the hyperactivity and over expression of FAS have been described in aggressive breast carcinomas [47]. Because FAS is the key enzyme involved in the FA synthesis, many studies have evaluated the effect of different FA (ω3 PUFA, CLA) on FAS activity by different nutritional or pharmacological approaches. For example docosahexaenoic acid (DHA) showed FAS inhibitory activity but to a lower level than CLA. Even closely related isomers may exert different inhibitory activity on FAS as exemplified by the difference between *Z*9,*E*11 and *E*10,*Z*12 isomers of the CLA [48].

**Figure 3:**
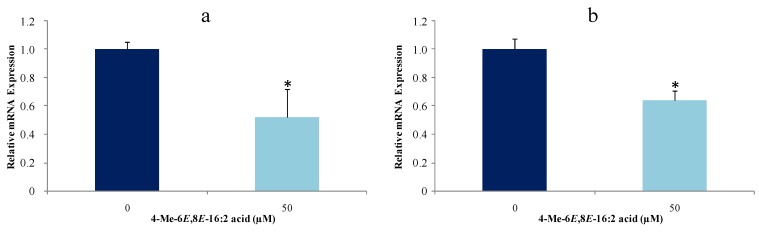
Effect of 4-Me-6*E*,8*E*-16:2 acid on gene expression of ACC (**a**) and FAS (**b**) of MCF-7 cancer cell line. MCF-7 cells were treated with 4-Me-6*E*,8*E*-16:2 acid at 50 μM for 24 h (*n* = 3 experiments in triplicates). * *p* < 0.05 *vs.* control (*t*-test).

Accordingly and because of the original source and structure of 4-Me-6*E*,8*E*-16:2 acid, it was further investigated whether this FA affects proliferation of mammary cancerous cells and mRNA expression of ACC and FAS. The effects of treatment with this CFA on expression of these two lipogenic enzymes on MCF-7 were evaluated by real time quantitative PCR. The concentration of 4-Me-6*E*,8*E*-16:2 acid used (50 μM, close to 50% of growth inhibition after 48 h), was in agreement with previous studies concerning CLA [16,49,50]. At 50 μM and after 24 h, gene expression was reduced for both enzymes (ACC, 50%, and FAS, 35%) (Figure 3).

The major observation of this study was that 4-Me-6*E*,8*E*-16:2 acid reduces ACC and FAS expression and inhibits their proliferation. However, its mechanism of action is not known. Some studies have reported that FAS inhibition in human breast cancer cells leads to accumulation of malonyl-CoA, which is an inhibitor of FA oxidation because it down-regulates CPT-1 (carnitine-palmitoyl transferase-1); an enzyme involved in mitochondrial FA uptake [51]. In addition, in breast and prostate cancers, similar changes have been found in the expression of ACC (high expression). This enzyme catalyzes the condensation of malonyl-CoA using acetyl-CoA and is considered as a rate-limiting enzyme of lipogenesis [52]. Silencing of ACC gene resulted in the inhibition of LNCaP prostate cancer cell line proliferation similarly to FAS suppression by RNAi technology. Overall, these data indicates the effect of 4-Me-6*E*,8*E*-16:2 acid on cancerous cell proliferation is mediated through its impact on lipid metabolism by down-regulating lipogenic enzymes ACC and FAS. More investigations are needed to clarify its role in the modulation of lipid metabolism.

## 3. Experimental Section

### 3.1. Clonostachys rosea Strain MMS1090

The fungal strain was isolated from sediments at Port du Bec, France, in 2002 and identified as *Clonostachys rosea* (Link: Fries) Schroers on the basis of molecular method by sequencing the internal transcribed spacers ITS1 and 2 and the 5.8S regions. The strain is conserved in the fungal collection of the laboratory MMS under the reference number MMS1090. Sequences of ITS1, ITS2 and 5.8S were deposited in GenBank under accession number KR011118 (National Center for Biotechnology Information, 2015).

### 3.2. Culture Media for OSMAC Approach

Petri dishes (10 cm diameter) were inoculated with conidia suspension (1 × 10^6^ cells/mL) in six marine-like solid media prepared with artificial sea water (Reef Crystals 36 g/L): DCA (Dextrose 40 g/L, enzymatic digest of Casein 10 g/L, Agar 15 g/L, Difco); MEA (Malt Extract 20 g/L, peptone 1 g/L, glucose 20 g/L, Agar 20 g/L; PDA (Potato extract 4 g/L, Dextrose 20 g/L, ZnSO_4_, 7H_2_O 0.01 g/L, CuSO_4_, 5H_2_O 0.005 g/L, Agar 15 g/L; YES (Yeast Extract 20 g/L, Sucrose 150 g/L, agar 20 g/L plus salts); MES (Mussel Extract 20 g/L, Sucrose 150 g/L, agar 20 g/L plus salts); CYA (Czapek concentrate 10 mL, Yeast extract 5 g/L, K_2_HPO_4_ 1 g/L, trace metal solution 1 mL, sucrose 30 g/L, Agar 15 g/L). Cultures were incubated in natural light during 10 days at 27 °C.

### 3.3. Lipid Extraction and Separation of Lipid Classes

For each culture media, biomass was removed with a scalpel and allowed to dry in an oven at 65 °C and weighing until a stable weight. Total lipids were extracted from the dried biomass during 2 h at room temperature by two-step CH_2_Cl_2_/MeOH extraction 2:1 then 1:2 (*v*/*v*). The resulting extracts were filtered, pooled and washed with distilled water (40% of total volume). Organic phase was then evaporated to dryness under vacuum providing crude total lipids (TL). TL are expressed in percentage of the total extracted dry biomass. Crude lipids were chromatographed on an open silica gel column with dichloromethane (triglycerides), acetone (glycolipids) and methanol (phospholipids) as successive eluents.

### 3.4. Preparation of Fatty Acid Methyl Esters and N-Acyl Pyrrolidides

FAME were prepared by saponification of crude lipids or lipid classes (1.5 h at 80 °C under reflux with KOH/EtOH 2 moL/L) followed by methylation of the free FA (40 min at 80 °C under reflux with 6% methanolic hydrogen chloride). NAP were prepared by direct treatment of the FAME with pyrrolidine/acetic acid (5:1 *v*/*v*) for 60 min at 85 °C under reflux.

### 3.5. Gas Chromatography-Mass Spectrometry Analysis

FAME and NAP were analyzed using a GC-MS instrument (Hewlett Packard HP 6890—GC System, Agilent Technologies, Santa Clara, CA, USA) linked to a mass detector (HP 6890—E.I. 70 eV) equipped with a SLB-5™ column (60 m × 0.25 mm × 0.25 μm). The carrier gas was helium at a flow rate of 1 mL/min. The temperatures of the injector and detector were respectively set at 250 °C and 280 °C. One microliter was injected in splitless mode. For FAME analysis, the column temperature was held at 170 °C for 4 min and programmed to 300 °C at 3 °C/min; for NAP, a bearing at 200 °C for 4 min, then 3 °C/min up to 310 °C and held for 20 min. The solvent delay was 9 min.

### 3.6. Production, Purification and Structural Elucidation of 4-Me-6,8-16:2 Fatty Acid

For production of 4-Me-6,8-16:2 FA, fungal cultures of *C. rosea* MMS 1090 were performed on DCA-containing Petri dishes (20 cm diameter, 125 mL) and incubated at 27 °C in natural light. After biomass extraction, total lipids were saponified, as described previously. Total FA were subjected to a urea inclusion step in order to obtain a fraction enriched in PUFA. Urea inclusion was prepared by heating FA mixtures and urea in MeOH under reflux for 2 h (1:2:0.01, *w*/*w*/*v*). After urea crystallization at room temperature, the urea-complex fraction enriched in saturated FA was removed and the filtrate enriched in PUFA and containing the 4-Me-6,8-16:2 acid was extracted with hexane and evaporated. The enriched fraction of 4-Me-6,8-16:2 FA was esterified under reflux (1 h) in MeOH/HCl 2% (10:1 *w*/*v*) and the 4-Me-6,8-16:2 methyl ester was then purified by a three-step silver ion chromatography method using two open columns followed by HPLC. Fraction purity was monitored by GC-MS analyses. Briefly, the enriched fraction of 4-Me-6,8-16:2 methyl ester was eluted from a first open column of silica gel impregnated with silver nitrate (Chromagel, 300 × 20 mm, 60Å, 40–63 μm, SDS, Peypin, France) using Hexane/Ethyl acetate 100:0 to 90:10 (*v*/*v*) as eluent. 4-Me-6,8-16:2 methyl ester was eluted with Hexane/Ethyl acetate 90:10 (*v*/*v*). This fraction was further subjected to a second open column (300 × 20 mm) using Cyclohexane/Ethyl acetate 98:2 to 90:10 (*v*/*v*) and eluted with Cyclohexane/Ethyl acetate 96.5:3.5 (*v*/*v*). Finally, the 4-Me-6,8-16:2 methyl ester was purified by an Agilent 1200 HPLC (Agilent Technologies, Santa Clara, CA, USA) on silver ion column (Varian Chrompack ChromSpher Lipids 250 × 4.6 mm, 5 μm, Agilent Technologies, Santa Clara, CA, USA ) with a mobile phase of Hexane/Acetonitrile 99.85:0.15 (*v*/*v*) at a constant flow rate of 1 mL/min. Detection was performed by UV at 215 nm. All data were acquired by HP ChemStation for LC.

Structural analysis was performed by infrared spectroscopy and ^1^H-NMR spectroscopy with CDCl_3_ to determine the configuration of the double bond. IR spectra were recorded on a PerkinElmer Spectrum 100 Series FT-IR spectrometer. ^1^H, ^13^C NMR spectra were recorded on a Bruker DXP 300 spectrometer at 300, 75 MHz, respectively and a Bruker AVANCE 400 MHz high resolution NMR spectrometer at 400, 100 MHz, respectively. Multiplicities are reported as singlet (s), doublet (d), doublet of doublets (dd), doublet of triplet (dt), triplet (t), multiplet (m).

### 3.7. Cell Viability Assay

Before pharmacological evaluation, 4-Me-6,8-16:2 acid was prepared by saponification of the purified FAME (1.5 h at 80 °C under reflux with KOH/EtOH 2 M).

Human breast cancer MCF-7 cells were purchased from the European Collection of Animal Cell Cultures (ECACC, Salisbury, UK). 3-(4,5-Dimethyl-2-thiazolyl)-2,5-diphenyltetrazolium bromide (MTT) and phorbol 12-myristate 13-acetate (PMA), were purchased from Sigma Aldrich (Saint Quentin Fallavier, France). 7-Aminoactinomycin D (7-AAD) was obtained from BD Biosciences (San Jose, CA, USA). Uptilight US Blot HRP substrate was from Interchim (Montlucon, France). All other reagents were purchased from Sigma Aldrich. MCF-7 cells were cultured at 37 °C in a humidified incubator with 5% CO_2_ in DMEM medium supplemented with 10% fetal bovine serum (FBS), 1% glutamine and 1% penicillin-streptomycin. Viability of MCF-7 was tested in 96-well plate at density 10^4^ cells per well in 200 μL of culture medium and allowed to adhere overnight. Then the seeding medium was removed and cells were treated with 4-Me-6,8-16:2 acid at 25 μM, 50 μM and 100 μM diluted in 0.1% BSA containing medium for 48 h. For MTT assay, 50 μL MTT (at 2.5 mg/mL) was added to each well at a final concentration of 833 μg/mL. The mixture was further incubated for 4 h, and the liquid in the wells was removed thereafter. Dimethyl sulfoxide (DMSO 200 μL) was then added to each well to solubilize the formazan product and the absorbance was read at 570 nm. The relative cell viability was expressed as a percentage of the control that was not treated with 4-Me-6,8-16:2 acid.

### 3.8. Gene Expression of Lipogenic Enzymes

MCF-7 cells were plated at a density of 5 × 10^5^ in a 6-well plate in 2 mL of culture medium and allowed to adhere overnight. Then the seeding medium was removed and cells were treated with 50 μM of 4-Me-6,8-16:2 acid diluted in 0.1% BSA-containing medium for 24 h at 37 °C. Total RNA was isolated by the TriZol Reagent (Invitrogen, Cergy Pontoise, France) following the manufacturer’s instructions. The mRNA (1 μg) was then reverse-transcribed into cDNA using Super-ScriptIII Reverse Transcriptase (Invitrogen, Waltham, MA, USA). An initial denaturation step for 5 min at 70 °C was followed by an elongation phase of 45 min at 50 °C. Quantitative-PCR was performed on a MyiQ2 Real-Time PCR Detection System (Bio-Rad, Marnes-la-Coquette, France) using SYBR Green Supermix. The PCR was carried out for 45 cycles of 95 °C for 30 s and 60 °C for 30 s. The fluorescence was read during the reaction, allowing continuous monitoring of the amount of PCR product. The results were normalized with the values observed for the gene expression of untreated cells.

## 4. Conclusions

This study of the marine-derived *Clonostachys rosea* MMS1090 confirm that this strain could be considered as oleaginous. This high lipid production is particularly obtained using high-sucrose-content culture media that lead to a high amount of TG (84% of total FA). The lipids are recognized to possess potent biotechnological interest.

Moreover this strain produces lipids composed of a rare CFA in high amounts (up to 23% of total FA) when grown on DCA medium. This unusual FA was identified as 4-Me-6*E*,8*E*-16:2 acid and is present mainly in the TG. To our knowledge this is the first study which refers to a branched CFA isolated from a marine-derived strain. In addition the two other isomers of this particular lipid were also detected.

This rare CFA reduces proliferation of mammary cancerous cells (MCF-7) at normal human plasma free FA concentration. This could be related to the down-regulation of lipogenic enzymes ACC and FAS. This preliminary *in vitro* study should be completed by *in vivo* studies to confirm the impact of this rare CFA in relation to regular dietary intake.

## Abbreviations

CFA: Conjugated fatty acid
FA: Fatty acid
FAME: Fatty acid methyl ester
GC-MS: Gas chromatography coupled to mass spectrometry
NAP: *N*-acyl pyrrolidide
OSMAC: One strain many compounds
PUFA: Polyunsaturated fatty acid
TL: Total lipids
TG: Triglycerides
ACC: Acetyl CoA carboxylase
FAS: Fatty acid synthase
